# How the deployment of visual attention modulates auditory distraction

**DOI:** 10.3758/s13414-019-01800-w

**Published:** 2019-07-09

**Authors:** John E. Marsh, Tom A. Campbell, François Vachon, Paul J. Taylor, Robert W. Hughes

**Affiliations:** 1 School of Psychology, University of Central Lancashire, Preston, PR1 2HE UK; 2 Department of Building, Energy, and Environmental Engineering, University of Gävle, Gävle, Sweden; 3 Faculty of Information Technology and Communication Sciences, Tampere University, Tampere, Finland; 4 Université Laval, Quebec City, Canada; 5 Department of Psychology, Royal Holloway, University of London, Egham, UK

**Keywords:** Selective attention, Cognitive control, Auditory distraction, Attentional capture, Interference by process

## Abstract

Classically, attentional selectivity has been conceptualized as a passive by-product of capacity limits on stimulus processing. Here, we examine the role of more active cognitive control processes in attentional selectivity, focusing on how distraction from task-irrelevant sound is modulated by levels of task engagement in a visually presented short-term memory task. Task engagement was varied by manipulating the load involved in the encoding of the (visually presented) to-be-remembered items. Using a list of Navon letters (where a large letter is composed of smaller, different-identity letters), participants were oriented to attend and serially recall the list of large letters (low encoding load) or to attend and serially recall the list of small letters (high encoding load). Attentional capture by a single deviant noise burst within a task-irrelevant tone sequence (the deviation effect) was eliminated under high encoding load (Experiment 1). However, distraction from a continuously changing sequence of tones (the changing-state effect) was immune to the influence of load (Experiment 2). This dissociation in the amenability of the deviation effect and the changing-state effect to cognitive control supports a duplex-mechanism over a unitary-mechanism account of auditory distraction in which the deviation effect is due to attentional capture whereas the changing-state effect reflects direct interference between the processing of the sound and processes involved in the focal task. That the changing-state effect survives high encoding load also goes against an alternative explanation of the attenuation of the deviation effect under high load in terms of the depletion of a limited perceptual resource that would result in diminished auditory processing.

Whilst there is little doubt that feature integration theory (Treisman & Gelade, 1980) was Anne Treisman’s single most influential contribution to psychological science, an earlier contribution that should not be overlooked is her *attenuation theory* of selective attention (Treisman, 1964a, 1964b, 1964c, 1964d; Treisman & Riley, 1969). This theory derived from the study of auditory attention phenomena that dominated early cognitive psychological research on attention, and which, more generally, was pivotal to the establishment of cognitivism as a viable paradigm for the scientific study of the mind (e.g., Broadbent, 1958; Cherry, 1955). Attenuation theory was a response to findings that were beginning to challenge the early-filter model proposed by Broadbent (1958). In his model, mental processing is limited at the stage at which stimuli are perceived (i.e., processed to a postcategorical level); hence, in this model, the selection (or ‘filtering through’) of input for privileged limited-capacity processing occurs ‘early’ in the stimulus-processing chain (*early selection*). However, to explain why unselected information could, on occasion, be perceived – such as when hearing one’s own name in an ‘unattended channel’ (Moray, 1959) – Treisman (1964a, 1964b, 1964c, 1964d) proposed a more nuanced view whereby the processing of unselected input is merely attenuated as opposed to being completely filtered out from postcategorical processing. As such, the theory proposed that unselected input could indeed be processed post-categorically, and hence selection could be ‘late’, depending on the amount of activation required for its perception. The theory was successful in accounting for a number of findings that the early-filter account could not (Treisman, 1969). More recent views (Lavie, 1995, 2000) share the fundamental assumption that an attentional selection mechanism is imposed by a capacity-limited stage within a linear stream of essentially discrete processing stages. Although, here, selection may be ‘early’ or ‘late’ depending on the perceptual demands of a given task.

Anne Treisman’s attenuation theory is a pivotal chapter in the early cognitive psychology of attention that focused on a supposed structural limitation on stimulus processing, out of which attentional selectivity emerges. However, it has since been argued that the classic early versus late selection debate was predicated on questionable premises (e.g., Allport, 1993). For example, the observation that unattended information fails to interfere with responding to task-relevant information in selective filtering paradigms (e.g., selective speech shadowing) implies neither that the unattended information is not processed, nor provides evidence for the existence of a limited stage of stimulus processing, since selective processing is being observed and limited capacity is merely an inference (Allport, 1989; Neumann, 1996). For example, the fact that postcategorical information that failed to cause interference when presented as irrelevant information can affect performance on the next trial if the same information is then task relevant indicates that the unattended postcategorical information had indeed been processed (Driver & Tipper, 1989).

The current investigation of selective attention has therefore been influenced by an alternative selection-for-action (as opposed to selection-for-processing) view that eschews the notion of a capacity-limited stage of processing (Allport, 1987; Neumann, 1996). Instead, this view proposes that limitations on performance result from specific functional constraints such as the typically sequential nature of motor action. On this approach, no limit on *processing* is necessarily assumed, and the theoretical question shifts to how the potential interference flowing from that processing is cognitively controlled (for an in-depth discussions, see, e.g., Allport, 1993; Neumann, 1996; Tipper, 2001). The present study builds in particular on recent research suggesting that the degree to which task-irrelevant auditory input impinges on task performance is dictated in part by the degree to which top-down control can be imposed to regulate the level of engagement in the focal task (Hughes, Hurlstone, Marsh, Vachon, & Jones, 2013; Marsh, Ljung, et al., 2018a; Marsh, Sörqvist, & Hughes, 2015a; Marsh, Yang, et al., 2018b). When a boost in engagement is promoted by an increase in task demands, or when engagement-control is relatively great in the first place due to a high trait capacity for executive control –as indicated by measures of working memory capacity [WMC]; (Engle & Kane, 2004)–, certain kinds of auditory distraction are attenuated if not eliminated (Hughes et al., 2013; Marsh, Sörqvist, Hodgetts, Beaman, & Jones, 2015a; Marsh, Sörqvist, & Hughes, 2015b; Marsh, Vachon, & Sörqvist, 2017; Sörqvist, 2010; though see Körner, Röer, Buchner, & Bell, 2017; Hughes and Marsh 2019).

The paradigm we have been using to study the cognitive controllability of auditory distraction involves presenting task-irrelevant sequences of sound during a visually presented short-term memory task (e.g., Colle & Welsh, 1976; D. M. Jones & Macken, 1993; Salamé & Baddeley, 1982). In a typical experiment in this paradigm, around six to eight verbal items (e.g., digits, words) are presented one-by-one on a screen at a rate of one or two items per second. Following the last item –or, in some studies, following a short retention interval–, the items are to be recalled in strict serial order (i.e., serial recall). The basic observation that makes this setting of interest to the study of selective attention is that serial recall performance is appreciably impaired if auditory distractors are presented during the task, even though participants are explicitly told to ignore them and are assured that they will not be quizzed as to the content of the sound (for reviews, see Hughes & Jones, 2001; Jones, 1999; Jones, Hughes, & Macken, 2010).

This line of research has suggested that serial recall is vulnerable to auditory distraction through two functionally distinct mechanisms (the *duplex-mechanism account*; e.g., Hughes, 2014; Hughes et al., 2013; Marsh, Yang, et al., 2018b). The first refers to when the preattentive, involuntary, processing of the sound interferes directly with a similar process deployed to perform the recall task. This *interference-by-process* mechanism is witnessed empirically in the form of the *changing-state effect*: When the distractors in the sequence ‘change in state’ from one to the next (e.g., “A, Q, J, G . . .”; or a sequence of tones changing in frequency) there is marked disruption, whereas there is far less, if any, disruption caused by a steady-state distractor (e.g., “A, A, A, A . . .”; or a repeating tone; Jones & Macken, 1993; Jones, Madden, & Miles, 1992). On the interference-by-process account (e.g., Jones & Tremblay, 2000), changing-state sound is particularly disruptive because the preattentive processing of the changes in the sound yields cues pertaining to order information whilst a steady-state sound is impoverished in terms of such information. The extraneous order information from a changing-state sound sequence interferes with the similar, but deliberate, process of rehearsing the to-be-remembered items in serial order in support of serial recall. The second mechanism in the duplex-mechanism account is *attentional diversion,* whereby the sound, rather than interfering specifically with a process involved in ongoing goal-related performance, draws selective attention away from the task goal (Hughes et al., 2013; Hughes, Vachon, & Jones, 2005, 2007; Marois & Vachon, 2018; Parmentier, 2014; Vachon, Labonté, & Marsh, 2017). This form of distraction has been studied mainly via the disruptive effect of a single sound that clearly deviates acoustically from the remainder of the sounds within the auditory sequence, such as one item presented in a male voice within a sequence of otherwise female-spoken items (Hughes et al., 2007, 2013).

Of particular interest in the present article is evidence suggesting that these two forms of distraction—interference by process and attentional diversion—signified in particular by the changing-state effect and the deviation effect, respectively—are differentially amenable to top-down cognitive control (Hughes, 2014; Hughes et al., 2013). Indeed, the dissociation in terms of their cognitive controllability is one of the key findings that underpins the argument that they are functionally distinct distraction phenomena. Specifically, if the difficulty of encoding the focal visual items is increased by embedding the items in static visual noise, then the deviation effect is eliminated, whereas the impact of changing-state sound remains unabated (Hughes et al., 2013). We have argued that the increase in encoding load leads to an active top-down boost in task engagement—designed to maintain performance levels in the face of the increased task demands (cf. Eggemeier, Crabtree, & LaPointe, 1983; Eggemeier & Stadler, 1984)—which serves to shield performance from the otherwise attention-diverting deviant. The changing-state effect, in contrast, is unaltered because there is no reason to think that increased task engagement would affect the interference-by-process mechanism underpinning that effect. That is, increased task engagement would not be expected to promote a shift away from the deliberate serial rehearsal process adopted to support serial recall. Independent evidence for this comes from the fact that serial recall performance itself is not impaired under high load. The key precondition for changing-state sounds to disrupt performance would therefore remain (see Hughes et al., 2013).

The differential impact of encoding load on the deviation effect and the changing-state effect has been influential in terms of theory development: This differential impact has not only provided support for a duplex-mechanism account of auditory distraction (Hughes, 2014), but also has undermined the viability of an alternative, unitary, account in which the changing-state effect and the deviation effect are both attributed to attentional diversion (e.g., Cowan, 1995; Röer, Bell, & Buchner, 2015). In this view, a sound that differs from its immediate predecessor (i.e., each sound in a changing-state sequence) draws attention away from the focal task more than a sound that is a repetition of that sound's predecessor, just as a deviant is more likely to divert attention compared to the preceding succession of nondeviating sounds (Bell, Röer, Lang, & Buchner, 2019a, 2019b; Körner et al., 2017). However, if it was the case that each change within a sound draws attention away from the focal task, akin to a deviant, then the changing-state effect should also be attenuated when attention needs to remain more steadfast on the focal task (i.e., under high encoding load).

However, the method used by Hughes et al. (2013) for manipulating task load was, arguably, less than optimal because the visual display was different across the two levels of load: In the high-load condition, the perceptual discriminability of each to-be-remembered item was reduced through the addition of static visual noise to each item. Such stimulus-degradation may impose data limitations on performance instead of, or in addition to, influencing central, attentional engagement, processes (Lavie & de Fockert, 2003; Norman & Bobrow, 1975). Indeed, the importance of matching stimulus displays across low and high task load conditions is underscored by prominent examples in the literature in which failures to do so have led to a great deal of ambiguity as to the correct interpretation of the effects of task load on distraction (Tsal & Benoni, 2010; Wilson, Muroi, & MacLeod, 2011). In the present study, therefore, we used a method in which the focal visual display was identical in the high and low encoding load conditions and where load was manipulated by orienting participants to one or another dimension of that display. Specifically, we capitalized for the first time in this context on hierarchical Navon letters in which a single ‘large’ letter (the *global* dimension) is made up of a number of small letters (the *local* dimension; Navon & Gopher, 1979). When the identity of the local and global letters are incongruent (e.g., an *S* made up of small *T*s) the global letter (*S*) interferes with trying to respond to (e.g., name) the identity of the local letter (*T*). In contrast, there is little or no interference from the local letter when having to respond to the global letter. It has been argued that directing attention and responding to the local elements of hierarchical stimuli is more effortful because the initial, unintentional, focusing of attention on the global dimension needs to be intentionally overcome to focus on the local level (Stoffer, 1994; see also Miller, 1981; Stablum, Ricci, Pavese, & Umiltà, 2001). We exploited such *asymmetric interference* here to manipulate encoding load in a serial recall task: In the low-load condition, participants were oriented to, and required to serially recall, the letters represented on the global dimension of seven Navon stimuli and to ignore the letters represented at the local level. Thus, in Fig. 1, the stimulus list to be recalled in the low-load condition would be ‘S, N, L, Q, Y, V, O’. In contrast, in the high-load condition participants were oriented to, and required to serially recall, the seven ‘local’ letters; thus in Fig. 1, the to-be-recalled list in this condition would be ‘T, U, K, I, B, Z, F’ (though note that in the experiments themselves, the identities of the letters to be recalled were the same in the low and high load conditions; see Method for Experiment 1 for details). The assumption was that the more demanding task of processing the local compared with global letters (e.g., Stoffer, 1994) would induce an active boost in task engagement to meet that increased demand (e.g., Eggemeier et al., 1983; Hughes et al., 2013). In turn, on the duplex-mechanism account, this upregulation of task-engagement will serve to shield performance against distraction by a deviant within the irrelevant sound sequence, but not shield it against interference produced by continuously changing sounds (i.e., the changing-state effect). On the unitary account, in which both the changing-state effect and deviation effect are due to attentional diversion (e.g., Bell et al., 2019a, 2019b), both distraction effects should be attenuated under high load.

**Fig. 1:**
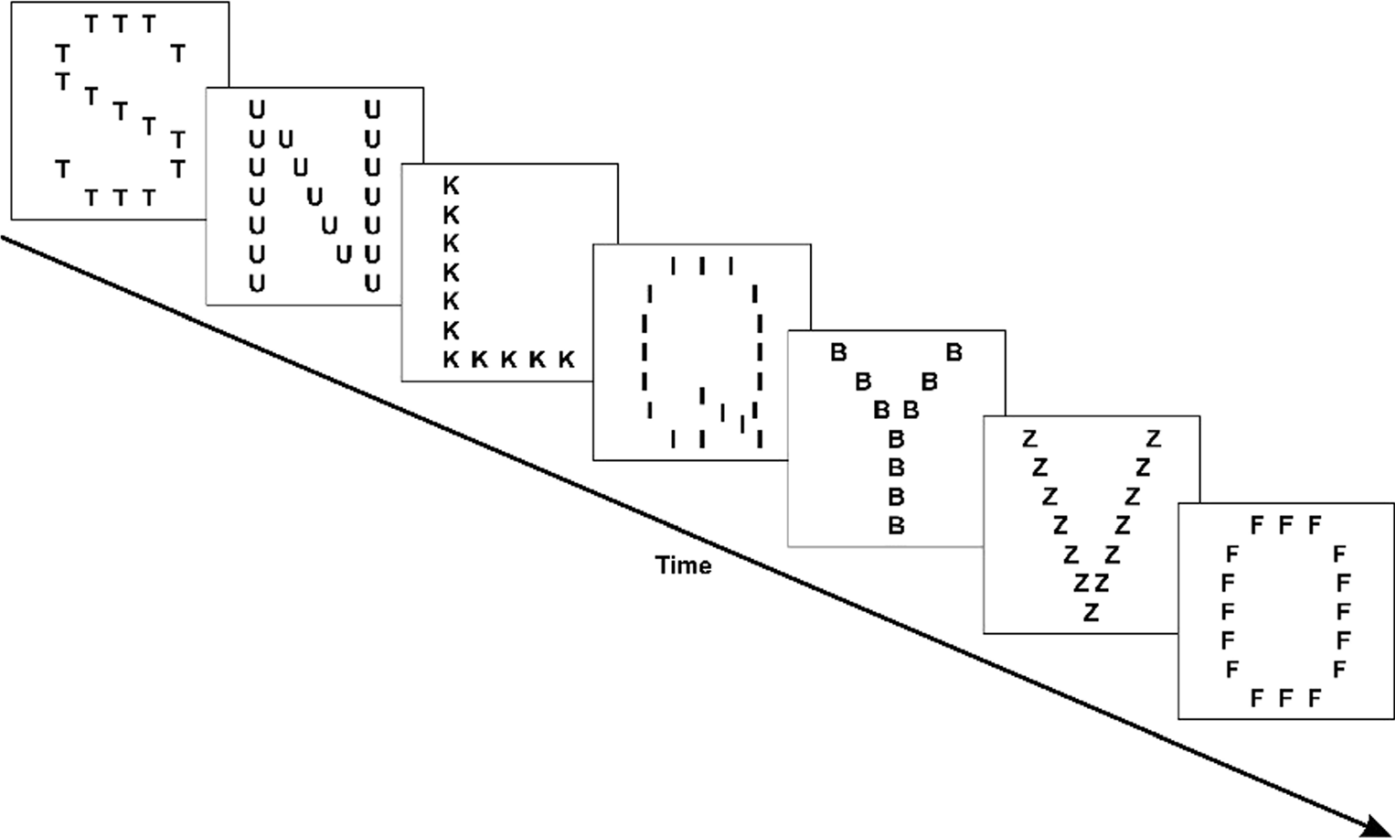
Schematic of a list of hierarchical Navon letters as used in both experiments reported in the present article. Participants were required either to focus on and recall the seven ‘global letters’ (low-load condition) or to focus on and recall the seven local letters (high-load condition).

The present manipulation of load will also afford a better test than hitherto of an alternative account of the attenuation of the deviation effect under high encoding load (Hughes et al., 2013)—namely, that based on *load theory* (Lavie, 2005). On our account, high encoding load promotes a top-down boost in task engagement that is assumed not to affect the processing of the predeviant sequence elements nor the detection of the deviant; rather, load influences performance following the detection of the deviant, but before attention would otherwise be shifted towards that shifted towards it (Hughes, 2014; Hughes et al., 2013). From the standpoint of load theory, in contrast, the attenuation would be explained in terms of the high perceptual load automatically preventing the processing of the sound sequence in which the deviant is embedded (e.g., Lavie, 2005). As with the unitary account of auditory distraction, but contrasting with the duplex-mechanism account, this load-theory-based account predicts that both types of auditory distraction should be attenuated under high load. That is, if high perceptual load suppresses the processing of task-irrelevant material, then the changing-state effect, and not only the deviation effect, should be attenuated under high load.

## Experiment 1

In Experiment 1, then, one group of participants were instructed to encode and serially recall the seven letters represented on the global dimension of seven Navon letters (low-load condition), while a second group were required to encode and recall the seven letters represented on the local dimension of the seven Navon letters (high-load condition). During the presentation of the memory stimuli, participants were exposed, on most trials, to a repeated (or ‘steady-state’) tone, but on a minority of trials were exposed instead to a steady-state sequence that contained a single burst of pink noise (the ‘deviant’; see also Marois, Marsh, & Vachon, 2019). We predicted that the disruptive effect on serial recall of the deviant sound would be attenuated in the high-load condition.

It is also worth highlighting that few studies have used nonverbal stimuli in the context of the deviation effect in short-term memory (Röer, Bell, & Buchner, 2014; Sörqvist, 2010) and none have examined whether the effect of load on the deviation effect generalizes to nonverbal deviations. Whilst there is no reason to expect the effect not to generalize beyond speech stimuli, it seemed of inherent value to establish this empirically.

### Method

#### Participants

Sixty students at the University of Gävle took part in return for two cinema tickets. All reported normal hearing and normal or corrected-to-normal vision.

#### Apparatus and materials

Fourteen letters were selected from the Swedish alphabet (excluding vowels with diacritics) and divided into two sets. The list of seven letters to be remembered (regardless of whether they were represented on the local or global dimension; i.e., regardless of load condition) was always ‘L, N, O, Q, S, V, Y’, while the set of letters that were to be ignored (again, regardless of load condition) was always ‘B, F, I, K, T, U, Z’. This approach ensured that what was to-be-recalled as well as what was displayed was identical across the two levels of load.

From these sets, hierarchical Navon letters were constructed such that each letter from the to-be-remembered set was paired with each letter from the to-be-ignored set. Within separate stimuli, each of the seven to-be-remembered letters was paired with each of the seven to-be-ignored letters, creating 49 unique combinations. Since each of the seven to-be-remembered letters could either comprise the large letter (global stimulus; with to-be-ignored letters comprising the constituent elements) or small letters (local stimulus; with the to-be-ignored letter comprising the whole), there were 98 Navon letters in total. For each trial, seven letters were sampled randomly from this hierarchical stimulus set with the constraint that each letter, whether from the to-be-remembered or to-be-ignored set, went without repetition in that sequence. The seven Navon stimuli on a given trial were presented one-at-a-time in the centre of the computer screen for 600 ms each, followed by an interstimulus interval of 300 ms. Letters were arranged in a pseudorandom order with the constraint that sequences of familiar letters or acronyms were avoided (e.g., ‘YO’, ‘SOY’). Moreover, there were no letters in successive trials, within the to-be-remembered or to-be-ignored sets, that shared the same within-sequence position.

During the presentation of the Navon stimuli, participants were presented with a task-irrelevant auditory sequence over their headphones. There were two types of irrelevant auditory sequence: ‘steady-state, no deviant’ and ‘steady-state with deviant’. In the steady-state condition, one of two sine tones, E4 or A4, was repeated 36 times. In the steady-state with deviant condition, a burst of pink noise replaced the 21st tone within the sequence, and this deviant occurred between presentation of the fourth and fifth Navon stimulus. Pink noise was chosen because it has been shown previously to be relatively potent at capturing attention (Wetzel, Buttelmann, Schieler, & Widmann, 2016). Tones were synthesized and edited to 100 ms with a 10-ms fade-in and fade-out. Each tone was recorded with 16-bit resolution at a sampling rate of 48 kHz, using Audacity Software. The irrelevant auditory sequence preceded the onset of the first to-be-remembered letter by 75 ms, and there was a 75-ms interstimulus interval between each tone (offset to onset). All auditory sequences were presented binaurally at approximately 65 dB(A) over Sennheiser HD-202 headphones. The experiment was executed on a PC running an E-Prime 2.0 program (Psychology Software Tools, Sharpsburg, PA, USA) that controlled stimulus presentation and recorded participant responses.

#### Design

A 2 × 2 mixed design was used, with auditory condition (no deviant, with deviant) as a within-participant factor and encoding load (low, high) as a between-participants factor, and the proportion of letters recalled in the correct serial position as the dependent variable. Regardless of load condition, participants undertook 45 trials (39 with a steady-state auditory sequence and six with a steady-state sequence containing a deviant sound). Again, regardless of load condition, the six with-deviant trials were Trials 6, 12, 18, 27, 34, and 43.

#### Procedure

The participants were tested individually in a sound-attenuated room in the presence of the experimenter. Participants were given standard written instructions concerning the serial recall task. They were told that there would be no repeats of any letter within a given list and that they should do their best to ignore sound that would be presented over the headphones because it was irrelevant to the task. Participants were not told anything about the presence of deviations within the sound sequences. Following the offset of the last Navon stimulus of a given list, participants were re-presented with the to-be-remembered letters they had just been asked to focus upon in an array wherein the position of each letter was determined randomly. The to-be-remembered letters were presented in this array as nonconflicting Navon stimuli (e.g., ‘*S*’ comprising small *S*s, ‘*Q*’ comprising small *Q*s). Beneath the array were seven response boxes arranged horizontally. Participants were required to click on the letters in the order that the to-be-remembered letters had appeared using a mouse-driven pointer. Once a letter was selected, a copy of that letter appeared in the response box. After the participant had made seven responses, there was a 3-s interval before the program prompted the participant to click on a ‘Begin Trial’ button to commence the next trial.

For the low-load group, the instructions explained that the to-be-remembered letters were represented on the global dimension of the Navon stimuli and that the letters represented on the local dimension were to be ignored. For the high-load group, the instructions explained instead that the to-be-remembered letters were those represented on the local dimension of the stimuli and that the global letters were to be ignored. Before taking part, participants were familiarized with which dimension of the stimuli they were to focus on and recall. This familiarization involved presenting four practice trials prior to undertaking the block of experimental trials. Each practice trial was accompanied by a steady-state sound sequence.

#### Analyses

For both experiments reported here, the data were analyzed using the analysis of variance (ANOVA) technique with an alpha level of .05. For each main and interaction effect, we report the classical *F* and *p* values along with an estimate of the effect size (${\upeta}_{\mathrm{p}}^2$) as well as the probability that the data favor the null hypothesis over the alternative hypothesis [*p*_BIC_(H_0_|D)], as computed using a Bayes factor analysis using Masson’s (2011) method.

### Results and discussion

**Fig. 2:**
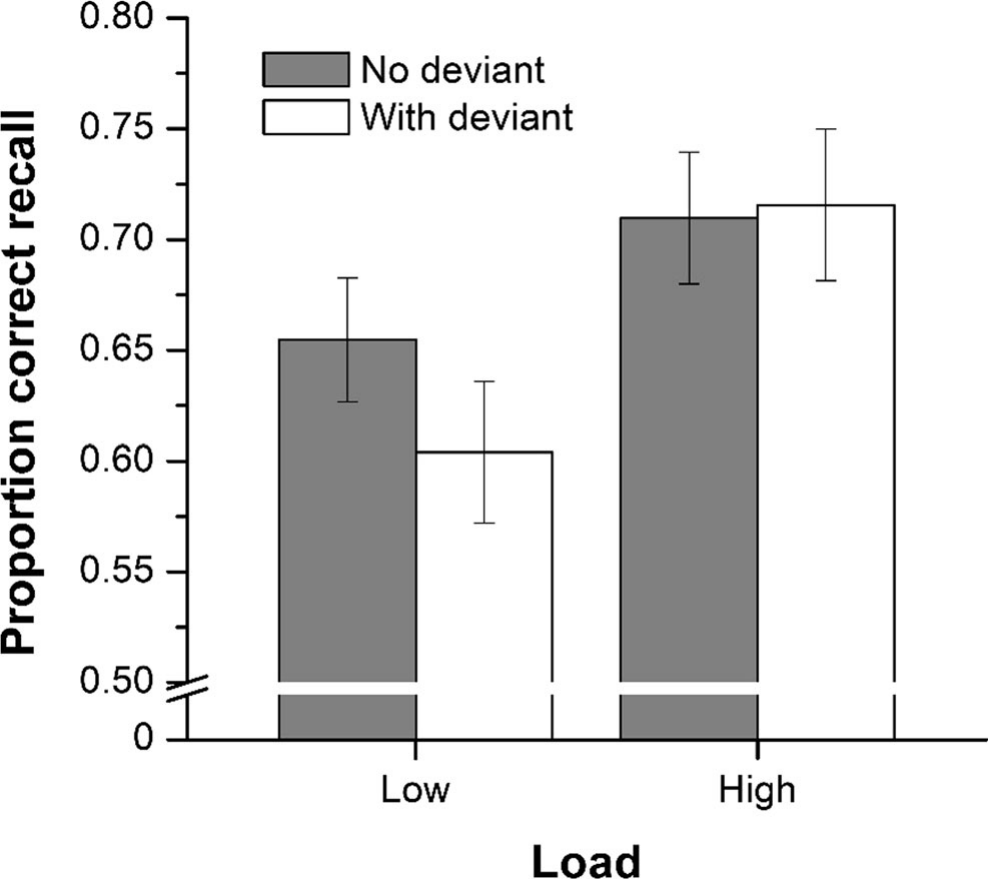
Proportion correct recall in the four conditions of Experiment 1 (*n* = 30 in low-load condition, *n* = 30 in high-load condition). Error bars represent the standard error of the mean

In all experiments reported within this article, the raw data were scored using the usual strict serial recall procedure whereby an item is only scored as correct if it is recalled in the same serial position as it was presented. Figure 2 shows serial recall performance—the proportion of correctly recalled items averaged across serial positions—for the four conditions of Experiment 1.

When participants were required to recall the global letters (low load), the deviation effect (e.g., Hughes et al., 2013; Hughes et al., 2007) was replicated: Serial recall was poorer in the presence of a deviant within the irrelevant sound sequence. This effect was, however, eliminated when participants were required to recall the local letters (high load). Of importance also is the fact that while high load eliminated the deviation effect, that load did not in-and-of-itself impair recall performance; indeed, recall performance in the high-load (no-deviant) condition was, if anything, better than in the low-load (no-deviant) condition. That serial recall was not impaired under high encoding load is consistent with our supposition that encoding of the local letters would very likely be impeded compared with the encoding of the global letters (Navon, 1977; Stablum et al., 2001), this impediment does not have any marked deleterious effect on the serial rehearsal of the items (cf. Experiment 2).

A 2 (auditory condition: with deviant, no deviant) × 2 (load: low, high) mixed ANOVA corroborated this impression of the data: The main effect of auditory condition was significant, *F*(1, 58) = 4.09, *MSE* = .004, *p* = .048, ${\upeta}_{\mathrm{p}}^2$= .066, *p*_BIC_(H_0_|D) = .499, and whilst the trend we noted for a facilitative effect of load per se did not quite reach the conventional level of significance, *F*(1, 58) = 3.89, *MSE* = .054, *p* = .053, ${\upeta}_{\mathrm{p}}^2$ = .06, *p*_BIC_(H_0_|D) = .532, there was, most importantly, a reliable interaction between auditory condition and load, *F*(1, 58) = 6.89, *MSE* = .004, *p* = .01, ${\upeta}_{\mathrm{p}}^2$= .11, *p*_BIC_(H_0_|D) = .237. A simple effects analysis of the interaction confirmed that there was a reliable deviation effect under low load, *p* < .005, 95% CI [.020, .082], *p*_BIC_(H_0_|D) = .079, but not under high load, *p* = .679, 95% CI [−.038, .024], *p*_BIC_(H_0_|D) = .823.

Experiment 1 demonstrated that the susceptibility of task performance to disruption by an auditory deviation is contingent on task-encoding load: When participants were required to identify and recall the ‘global letters’ in a list of hierarchical Navon letters, recall was impaired in the presence of a deviation in the irrelevant sound sequence but when required to identify and recall the ‘local letters’, the deviant was no longer disruptive. We suggest that the high encoding load of the recall-local condition induced an upregulation of task engagement such that attentional diversion from the task at hand became less likely (cf. Hughes et al., 2013). The experiment also established that the effect of encoding load on the deviation effect generalizes to nonspeech sounds.

We turn now in Experiment 2 to examine the opposing predictions of the duplex-mechanism account (Hughes, 2014; Hughes et al., 2013) and the unitary account (Bell et al., 2019a, 2019b; Röer et al., 2015) of auditory distraction with respect to the relation between encoding load and the deleterious impact on serial recall of a continuously changing sound sequence compared to a steady-state sound. This experiment will also address whether load theory (Lavie, 2005) could provide an alternative and more parsimonious account of the load effect shown in Experiment 1.

## Experiment 2

Given that high encoding load shields against attentional diversion by a deviant, and given that the unitary account attributes the changing-state effect, and not just the deviation effect, to attentional diversion, then the same increase in load should also attenuate the changing-state effect. In contrast, the duplex-mechanism account posits that the changing-state effect is caused instead by a competition between the obligatory processing of the order of the elements in a changing-state irrelevant sound sequence and the deliberate ordering (via vocal-motor serial rehearsal) of the to-be-remembered items (Hughes, 2014). According to this account, the changing-state effect should not be affected by encoding load because there is no reason to think that, even though stimulus encoding would be slower under high load, participants would not ultimately serially rehearse the items. Indeed, the fact that there was no deleterious main effect of load on serial recall performance provides support for this assumption.

Experiment 2 will also speak to an alternative theoretical account of the results of Experiment 1: that the attenuation of distraction was a passive side effect of increased perceptual load (Lavie, 2005; Lavie & Tsal, 1994) rather than the result of a dynamic top-down boost in task engagement (Hughes, 2014; Hughes et al., 2013). The load theory of attention (Lavie, 2005) adheres to the structuralist notion of a fixed processing-capacity limit (cf. Broadbent, 1958; Treisman, 1964a, 1964b, 1964c, 1964d), but whether selection is ‘early’ or ‘late’ is a variable function of the extent to which that capacity is exhausted by the perceptual demands of the focal task: If perceptual capacity is used up by the focal task, then there is no spare capacity to perceive any nontask, and hence potentially distracting, stimuli (hence, in this situation, selection is ‘early’). If, however, the perceptual demands of the focal task are relatively low, then perceptual capacity inevitably ‘spills over’ to process any nontask stimuli, thereby leaving task performance vulnerable to distraction by those stimuli (hence, in this situation, selection is ‘late’). Of particular relevance to the current cross-modal (auditory-visual) setting, Molloy, Griffiths, Chait, and Lavie (2015) found that when the perceptual load in a visual search task was high due to a similarity (compared with a dissimilarity) between the target and nontargets, there was a failure to perceive task-irrelevant tones, which was accompanied by reduced auditory evoked potentials; they thus concluded that “temporary depletion of shared capacity in perceptually demanding visual tasks leads to a momentary reduction in sensory processing of auditory stimuli” (p. 16046).

Our manipulation of task load in the present experiments appears to fit well with what constitutes a manipulation of perceptual load: “Increased perceptual load means that either the number of different-identity items that need to be perceived is increased, or that *for the same number of items perceptual identification is more demanding on attention*” (Lavie, 2005, p. 75, emphasis added). For example, Brand-D’Abrescia and Lavie (2007) described the requirement to search for a target letter within a visually presented nonword as imposing greater perceptual load than searching for the target letter in a word. Indeed, it would be difficult to class the present load manipulation as anything other than one of perceptual load (in the parlance of load theory) because increases in the other two kinds of load to which the theory has addressed itself—cognitive load (e.g., Lavie, 2010) and sensory load (Lavie & de Fockert, 2003)—are predicted to exacerbate rather than attenuate distraction (Lavie, 2005). Load theory could, then, readily explain the results of Experiment 1: The requirement to encode the local (compared with global) dimension of the Navon stimuli left no spare perceptual capacity to perceive the auditory distractor sequence. As such, a deviation within that sound sequence would not be detected and hence could not capture attention. If, however, the higher perceptual load of the ‘recall local letters’ condition did indeed lead to the suppression of the perception of the irrelevant sound sequence, then *any* form of distraction produced by that sequence should, presumably, be attenuated. In particular, load theory predicts, in contrast to the dynamic task-engagement account (Hughes et al., 2013), that the high ‘perceptual’ load should attenuate the usual disruption of serial recall by continuously changing-state sound compared with a steady-state sound (Jones & Macken, 1993; Jones et al., 1992).

### Method

Most aspects of the method of Experiment 2 were the same as Experiment 1, and any differences are highlighted below.

#### Participants

Thirty participants recruited from the campus community at the University of Central Lancashire took part in the experiment in return for a small honorarium. All reported normal hearing and normal or corrected-to-normal vision.

#### Apparatus and materials

Irrelevant auditory sequences comprised a repeated sine tone (steady state) or two alternating sine tones that differed from one another by 5 semitones (changing state). Each sequence comprised 36 tones. The tones of pitch E4 and A4 from Experiment 1 were used. Following Jones, Alford, Bridges, Tremblay, and Macken (1999) and Experiment 1, each tone had a duration of 100 ms, and there was a 75-ms intertone interval. In contrast to Experiment 1, encoding load was manipulated within participants. There were 32 trials in the low-load condition and 32 trials in the high-load condition. The trials were pseudorandomised such that trials comprising the same-tone sequence were not presented more than twice in succession. Each block of trials began with four trials in silence so that participants could familiarize themselves with the task demands, especially the different requirements in the two load conditions. The order in which the separate low-load and high-load blocks of trials were undertaken was counterbalanced across participants.

#### Design and procedure

The experiment had a 2 × 2 repeated-measures design, with auditory condition (steady state, changing state) and encoding load (low, high) as the independent variables, and serial recall performance as the dependent variable.

### Results and discussion

**Fig. 3:**
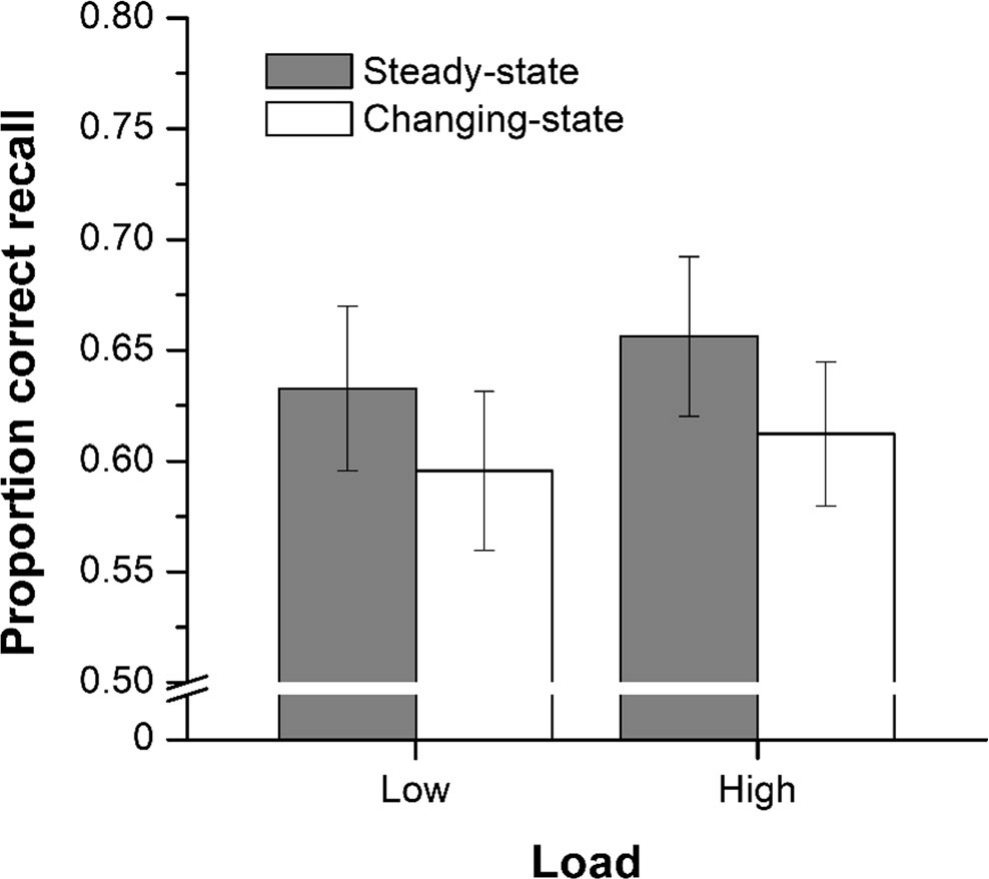
Proportion correct recall in the four conditions of Experiment 2 (*n* = 30). Error bars represent the standard error of the mean

Figure 3 shows serial recall performance averaged across serial positions for the four conditions in Experiment 2. It is clear that serial recall was poorer in the changing-state compared with the steady-state condition (i.e., the changing-state effect), and there is no evidence that this effect, unlike the deviation effect examined in Experiment 1, was modulated by load. A preliminary investigation revealed that there was no effect of block order, *F*(1, 28) = .00, *MSE* = .14, *p* = .99, ${\upeta}_{\mathrm{p}}^2$ = .00, *p*_BIC_(H_0_|D) = .154, and that block order did not interact with the auditory condition, *F*(1, 28) = .55, *MSE* = .01, *p* = .46, ${\upeta}_{\mathrm{p}}^2$ = .02, *p*_BIC_(H_0_|D) = .780, encoding load, *F*(1, 28) = .91, *MSE* = .004, *p* = .35, ${\upeta}_{\mathrm{p}}^2$ = .03, *p*_BIC_(H_0_|D) = .196, or auditory condition and encoding load, *F*(1, 28) = .37, *MSE* = .002, *p* = .55, ${\upeta}_{\mathrm{p}}^2$ = .01, *p*_BIC_(H_0_|D) = .194. Any effects and interactions involving block order were thus small and nonsignificant. Thereafter block order was removed from analyses.

A 2 (auditory condition) × 2 (encoding load) ANOVA revealed a main effect of auditory condition, *F*(1, 29) = 13.54, *MSE* = .004, *p* = .001, ${\upeta}_{\mathrm{p}}^2$ = .318, *p*_BIC_(H_0_|D) = .017. There was no main effect of encoding load, *F*(1, 29) = 1.14, *MSE* = .011, *p* = .295, ${\upeta}_{\mathrm{p}}^2$ = .038, *p*_BIC_(H_0_|D) = .756 (and the slight trend for a difference was towards better recall under high load), and there was no interaction between auditory condition and encoding load, *F*(1, 29) = 0.197, *MSE* = .002, *p* = .661, ${\upeta}_{\mathrm{p}}^2$ = .007, *p*_BIC_(H_0_|D) = .832.

The results of Experiment 2, when considered together with those of Experiment 1, support the duplex-mechanism account: The same increase in load that eliminated the deviation effect had no impact on the changing-state effect. This finding is consistent with the contention—supported by several lines of converging evidence (e.g., Hughes, 2014; Marois et al., 2019)—that the changing-state effect is not due to attentional disengagement from the task but due to interference between the obligatory processing of the sound and processes involved in performing the task. This dissociation also goes against a unitary account of auditory distraction in which both effects result from attentional diversion (Bell et al., 2019a, 2019b; Röer et al., 2015). Finally, the dissociation undermines the viability of an alternative possible account of the attenuation of the deviation effect in Experiment 1 whereby the high perceptual load (cf. load theory; Lavie, 2005) imposed by the requirement to recall the local compared with the global letters automatically blocks the processing of the sound sequence. If this were the case, the impact of changing-state compared with steady-state sound should also have been attenuated.

## General discussion

In the present experiments, we manipulated task encoding load in a visual–verbal serial recall task in a novel way by orienting participants to one or another dimension of a list of hierarchical Navon stimuli. In the low-load condition, participants were to recall the ‘large letter’ represented on the global dimension of the Navon stimuli, while in the high-load condition, they were to recall the ‘small letter’ represented on the local dimension. This approach meant that we could for the first time examine any influence of encoding load on auditory distraction without altering the actual visual stimuli across different levels of load (cf. e.g., Halin, Marsh, & Sörqvist, 2015; Hughes et al., 2013; Marsh, Ljung, et al., 2018a; Marsh, Yang, et al., 2018b) which has, in other settings, complicated the interpretation of load effects on selective attention (Benoni & Tsal, 2013; Tsal & Benoni, 2010; Wilson et al., 2011). Experiment 1 showed that high load eliminated the otherwise disruptive effect of a deviant sound within an irrelevant sound sequence (e.g., Hughes et al., 2005) while Experiment 2 showed that the disruptive effect of a changing- compared to steady-state sequence (cf. Jones et al., 1992) was unaltered by that same increased in load.

The findings provide further evidence for the duplex-mechanism account of auditory distraction (Hughes, 2014; Hughes et al., 2013). In this view, the deviation effect results from attentional diversion, a disengagement from focal task processing that precedes an assessment of the significance of an event that has violated expectations based on the prevailing auditory scene (e.g., Schröger, 1997; Vachon, Hughes, & Jones, 2012). From this standpoint, high encoding load leads to a top-down boost in task engagement that is designed to maintain focal-task performance levels (cf. Eggemeier et al., 1983; Eggemeier & Stadler, 1984) such that the disengagement in response to the deviant is circumvented. An important assumption of the task-engagement account is that the sound sequence is processed, and the deviant detected, as normal under high encoding load but that the attentional response to the deviant is suppressed (Hughes, 2014; Marsh et al., 2018a,b). This aspect of the account distinguishes it from an alternative account of the elimination of the deviation effect under load that we suggested could be drawn from load theory (e.g., Lavie, 2005). Here, high encoding load (or ‘perceptual load’ in the parlance of this theory) would exhaust perceptual capacity that would otherwise be applied, automatically, to process nontask stimuli. Thus, in this view, the sensory processing of nontask stimuli is attenuated or blocked under high perceptual load. The results of Experiment 1, then, in-and-of-themselves, do not adjudicate between the task-engagement account and load theory. However, the results of Experiment 2 lead us to favour the task-engagement account of the results of Experiment 1: If the high encoding load implemented here affects an attentional response to (otherwise fully perceived) irrelevant stimuli (task-engagement account) rather than affects the perceptual processing of the sound per se (Lavie, 2005), then there is no reason to expect the changing-state effect to be modulated, because this effect is not due to an attentional capture response.

That high encoding load eliminates the deviation effect but not the changing-state effect coheres with the conceptually similar dissociation reported by Hughes et al. (2013), who used sensory degradation to increase encoding load. But how is the deviation effect attenuated? We argue that our data support a ‘late-blocking’ view (e.g., Hughes et al., 2013; Marsh et al., 2018) over the ‘early-filtering’ view offered by the perceptual load component of load theory (Lavie, 1995, 2000; for similar views, see de Fockert & Theeuwes, 2012; Sörqvist, Stenfelt, & Rönnberg, 2012). The late-blocking view proposes that deviants within irrelevant sound sequences are equally detectable regardless of task engagement but that stronger task engagement circumvents the actual switch of attention to the deviant or cuts short the evaluation of the event if attention is switched to the deviant (see also SanMiguel, Corral, & Escera, 2008), permitting a speedier resumption of the focal activity (e.g., Parmentier, Elford, Escera, Andrés, & San Miguel, 2008). One possibility is that increased task engagement is synonymous with the strengthening of the activation of task representations related to the focal activity (i.e., the task set) such as the task-goal, what rules need to be followed to achieve that goal, what strategies are best to deploy, predictions about what task-relevant stimuli might occur, and the representations of such stimuli once they have occurred (cf. Desimone & Duncan, 1995). When the deviant occurs, these task-representations serve as cues to ‘stay on task’, shielding against the potential distraction produced by the detection of change in the auditory environment.

This late-blocking view is also supported by electrophysiological evidence that increasing encoding load reduces the involuntary attentional orienting toward the deviant sound—hence the amount of distraction—without altering the early detection of that deviating event. Some researchers have assessed the impact of encoding load on auditory distraction using two components of the event-related potential elicited by deviant sounds: the mismatch negativity (MMN), reflecting the preattentive detection of acoustical irregularities, and the P3a, indexing the orienting of attention. The manipulation of encoding load varied across studies. A high encoding load could be induced by postponing a perceptual judgment on a given stimulus until the presentation of the next stimulus (Berti & Schröger, 2003), requiring the comparison of digits across two consecutive stimuli rather than within a single stimulus (SanMiguel et al., 2008), or increasing the number of tracked targets in a visual tracking task (Zhang, Chang, Yuan, Zhang, & He, 2006). These studies revealed that under high load, as the amplitude of the P3a was reduced, so was the amount of behavioural distraction. However, increasing encoding load failed to diminish the amplitude of the MMN. Such a pattern of results suggests that the modulation of exogenous attention mechanisms by top-down control does not take place at the stage of initial deviant detection but rather at the subsequent stage of the orienting response, consistent with our late-blocking view.

How might the late-blocking view explain the finding of reliable individual differences in susceptibility to the deviation effect, but not the changing-state effect, that are accounted for by differences in WMC? We suggest that stable individual differences in the capacity for task activation as well as moment-to-moment variation within a given individual (possibly associated with WMC; Engle & Kane, 2004) can determine the representation of task-set, thus accounting for the greater susceptibility to the deviation effect of individuals with lower WMC (Hughes et al., 2013; Marsh et al., 2017; Sörqvist, 2010; though see Körner et al., 2017). This task-engagement view asserts that individual differences in WMC reflect individuals’ trait capacity for focal-task engagement: High WMC enables people to reach higher states of focal task engagement, a by-product of which is better distractor shielding (Sörqvist & Marsh, 2015). This view of WMC—that it reflects trait capacity for focal-task engagement, and that high focal-task engagement shields against distraction as a by-product—differs in important ways from the executive attention view (Engle, 2002), wherein WMC is regarded as the capacity to avoid distraction. Whilst the executive attention view posits that high-WMC individuals are less susceptible to distraction as a result of their having more resources available to combat distraction (an active mechanism), the focal-task engagement view explains the shielding effect as a passive side effect of a more steadfast locus of attention. We favour the late-blocking view over the early-filtering view (Lavie, 1995, 2000), the latter assuming that high encoding load attenuates the processing of sound sequences as a whole, thereby impairing the detectability of deviants and making one ‘deaf’ to environmental change. This would appear to be maladaptive since the organism would be unresponsive to deviant events just at the wrong time: when it is already dealing with a threatening and hence ‘high-load’ situation. The late-blocking view wherein environmental changes that could signify potential threats to survival are still registered but the actual switch of attention to the change (e.g., deviant) is prevented, or its evaluation is cut short, gels better with the notion that the auditory system serves as an early-warning system that signals environmental danger (or opportunity). As Berti and Schröger (2003) note:

This limited control over involuntary attention switches at stages subsequent to the initial change detection system most probably helps to focus on the task-relevant information under higher situational demands without losing the ability to scan the environment preattentively. (p. 1122)

Early cognitive research on selective attention proposed that a need for attentional selectivity was imposed by a structural processing limitation within the cognitive system. For example, in the context of the early filter view (Broadbent, 1958), a filter protected the limited-capacity perceptual channel (and thus the cognitive system) from overload. Anne Treisman’s (e.g., 1964a, 1969) seminal work on auditory attention challenged the rigidity of this filter, suggesting that the filter merely attenuates the processing of unattended stimuli, thereby allowing information—depending on its existing threshold of activation—to permeate the filter. The influence of these structuralist accounts can still be observed in modern approaches wherein a limited resource or set of resources determines attentional selectivity (e.g., Lavie, 1995, 2005). In these approaches, interference produced by task-irrelevant information is used as a ‘yardstick’ of where in the system stimulus processing is limited, yielding early or late selection (e.g., Broadbent, 1958; Treisman, 1964a, b, c, d). Some of our recent research into attention and distraction (present study; Hughes & Marsh, 2017; Marsh et al., 2018a, b; Vachon et al., 2012), however, has been allied to the selection-for-action view (Allport, 1987, 1993; Neumann, 1996), wherein interference is construed as a by-product of the action of processes and mechanisms that solve selection-for-action problems. In this view, there is no linear monotonic sequence of discrete processing stages, one of which is severely capacity limited. Rather, the limitation on performance results from having to populate a motor plan with only one out of potentially several competing perceptual objects because motor action is typically serial and the number of effectors scarce (e.g., we only have one vocal tract that can only produce one item at a time). Thus, rather than reflecting an architectural bottleneck, attentional selectivity is an achievement of dynamic control processes—such as those involved in determining levels of focal-task engagement—that ensure that only one of several possibly fully processed streams of information assume the control of ongoing behaviour (Hughes & Marsh, 2017; Neumann, 1996).

### Author note

We would like to thank Sebastian Arnström and Tanya N. Joseph for data collection. John E. Marsh’s contribution to this article was supported by a grant from the Swedish Research Council (2015-01116) awarded to Patrik Sörqvist and John Marsh.
